# The Hookworm, the Antidote to Effort; the Germ of *Dolce Far Niente*, the South’s “Bromide”

**Published:** 1911-06

**Authors:** 

**Affiliations:** New York City


For Texas Medical Journal.

### The Hookworm, the Antidote to Effort; the Germ of *Dolce Far Niente*, the South's "Bromide"

BY A SOUTHERN NEW YORKER.

From early colonial days the South has been noted for the ease and ennui of her citizens. The people of the South descended from the cavaliers—whose forbears were men and women of ease and comfort. From the earliest period of the permanent settlement, this section of the county has been noted for its geniality and gentleness. From the day when the rhetorical suggestion about the wonderful hospitality of the South took its rightful place with kindred aphorisms, we of the South have extended ardent welcome to every man with money. Having nothing to enliven us except the nebulus notion that we might separate him from some of his gold.

Into this Eden crept one Rockefeller with a scheme to filch from this happy and contented section of the moral vineyard, "its hallowed hookworm," to quote the words of Hon. Caruthers Ewing in an after-dinner speech in Washington, D. C., not long since. He said:

"I have brooded much over this atrocity, and have come to regard it as an act wanton and unpardonable, and he brands 'John

D.' as lost to all social obligations, and with a craven heart on mischief bent.

"Reverting to Mr. Rockefeller's invasion of our traditional rights—and, while the South is not so particular about its rights, it is foolish about its tradition—I say to you that it was not because he loved or lusted for the worm, but because of his insatiate desire to crush something.

"Just why he should have turned his evil eye upon the South and proposed to annihilate nature's sure antidote to effort, I do not know. We never sold any coal oil. And I hope the fact will obtrude itself upon his conscience, before the final tragedy, and cause the still, small voice—if there are any still, small voices left—to beseech him: 'Oh, Mister, spare that worm!'

"If he met that hookworm in the fair field of light, competing with it for supremacy—man or worm—our rage and resentment would not have mounted so high. But he subsidized scientists and marshaled the myrmidons of medicine, applied to that worm the hideous epithet 'Uncinaria,' and with no search warrant from on high proposes to forage our forms and probe our persons, and I tell you tonight that the peace of universal man is placed in peril.

"I do not speak for the worm, but for the world. That worm makes a man subside, it stops the soaring purpose and makes the wild wave of tumult be as the still waters. If there was ever a time in human history when the hookworm could serve a God-ordained end, it is today. Dr. Stiles, one of the Rockefeller's Mamelukes of Medicine, says that just one little worm renders a man incapable of performing any great exertion. The crying need of the hour, then, is that worm."

If one hookworm had been placed within Geo. H. Simmons before he left the sage brush of the wild and woolly West, before he made the trade with the Chicago diploma mill, before he became a regular through irregular methods, before he became "ye editor of the great Medical Octopus," his grasp on that gigantic medical trust would not have been equal to his frenzy, and the present cup of medical subsidence would verily have passed from us. No mature man within the ranks of honest medicine can truthfully say that this small object of Rockefeller's wealth, if well and wisely used, might not be a blessing to humanity, though it could not destroy the walking delegate's willingness to go into ye byways and hedges and compel the lingering medicos to come into the fold or be stigmatized as scabs—unfit to rub elbows with the elect.

There is one citizen in the Windy City whose system should be saturated with hookworms, if the serenity of the medical world is to be assured. Think of the storms, cyclones, etc., that might have been averted and the tranquility that would pervade the annual meetings of the A. M. A. if this mighty man of medicine had some hookworms. We would suggest that Lydston, the hard-hitter, supply himself with a liberal quantity and give him a lethal dose; it might be more effectual than printers' ink. No doubt a coalescing of ye Editor of the Octopus and the Uncinaria might produce a result that would thrill the medical throne of the great founder of medical ethics, and shake his world so hard that he would hie himself to the scenes of his early advertising days and return to his former vocation of writing death certificates.

No man with the hookworm reposing in him ever captured the editorship of a great medical journal; no such man ever braved the bolts of the wrath of Lydston, or captured the empire of medicine and pharmacy, or taught insurging doctors how a real insurgent should insurge. No man with a hookworm ever left his fatherland, and, by quackery, ascended the throne of American medicine and wielded the scepter of power so strenously that the medical populace feel like the fellow whose pet lion wandered away—lonesome and less scared. Rather than flee, as did the Benjamite, to the Rock of Rimmon, let us meet and greet him with our most virile hookworm and, with bowed head and bated breath, watch the decline and fall of the Dynamite Dynasty.

"The time will come in this distracted land when the essence of the hookworm will be more valuable than radium—when it can accomplish more good than a senatorial investigation. When it will be more potent in allaying the anxiety of the medical world than George H. Simmons in arousing it, and may not every lover of his kind hope that, under the gentle ministration of that worm, the walking delegate and those of his kind will sink into innocuous desuetude.

"The people of the South want their hookworms; the people of the North and East need them. No thoughtful, patriotic citizen of this republic can view with unconcern a scheme to add to human activities. The tide of affairs is moving like a tempest on the face of the waters. And the hookworm is nature's last warning—'Stop, Look, and Listen.'"

New York City, April, 1911. W. E. F.

